# Regulation of Rainbow Trout Muscle Myofibrillar Networks by Non-Enzymatic Bioactive Compounds from *Chaenomeles speciosa*

**DOI:** 10.3390/gels12090845

**Published:** 2026-09-16

**Authors:** Rui Chai, Yidian Li, Zibo Song, Xuejiao Wang, Zhenna Zhang, Chaofan Guo, Junjie Yi

**Affiliations:** 1 Faculty of Food Science and Engineering, Kunming University of Science and Technology, Kunming 650500, China; 2 Innovation Research Group on Fruit and Vegetable Processing and Nutritional Health, Kunming 650500, China; 3 Yunnan Key Laboratory of Plateau Food Intelligent Processing, Kunming 650500, China; 4 Yunnan International Joint Laboratory of Green Food Processing, Kunming 650500, China; 5 Yunnan Provincial Key Laboratory of Applied Technology for Special Forest Fruits, Yuxi 653100, China; 6 Yunnan Maoduoli Group Food Co., Ltd., Yuxi 653100, China; 7 College of Food Science and Engineering, Northwest A&F University, Yangling 712100, China

**Keywords:** processing treatments, myofibrillar conformation, small bioactive molecules, water-holding capacity, thiol–disulfide balance, volatile compounds

## Abstract

During marination and boiling processes, structural rearrangement of muscle proteins plays an important role in determining the texture, water retention, and flavor quality of fish products. This study investigated the effects of *Chaenomeles speciosa* aqueous extract (SE), rich in non-enzymatic bioactive compounds, on the myofibrillar networks of rainbow trout muscle. The effects of SE on rainbow trout muscle quality were evaluated under marination and boiling conditions. SE treatment improved muscle tenderness, water-holding capacity, and flavor characteristics under both processing conditions. The hardness of boiled muscle decreased from 15.06 N in the control group to 6.03–8.29 N after SE treatment, while shear force decreased by 19–31% and 18–32% under marination and boiling conditions, respectively. The water-holding capacity of boiled muscle reached approximately 78–79% after treatment with SE at 80 mg/mL. Structural analyses, including low-field nuclear magnetic resonance and scanning electron microscopy, suggested that SE treatment was associated with changes in water distribution and the formation of a relatively looser and more flexible myofibrillar network, which may be related to alterations in protein conformation, surface hydrophobicity, and thiol–disulfide balance. Furthermore, low-to-moderate SE concentrations reduced key fishy odor-related compounds, with octanal decreasing by 70.2% in the SE-80-B group. Importantly, these effects were still observed after boiling, when protease activity was undetectable, suggesting that heat-stable non-enzymatic components of SE may play a role in modifying muscle protein organization beyond the effects of acidification or proteolysis. Overall, SE may serve as a potential natural ingredient for improving the processing quality of rainbow trout products.

## 1. Introduction

Plant-derived extracts have attracted increasing attention in aquatic food processing because their bioactive constituents can improve product quality through antioxidant activity and interactions with food proteins. Plant phenolic compounds have been reported to inhibit oxidation and modulate protein conformation and functional properties in muscle foods [1,2,3]. In contrast, plant proteases such as papain improve tenderness mainly through enzymatic degradation of myofibrillar and connective-tissue proteins [4,5]. However, whether non-enzymatic constituents from plant extracts can regulate fish-muscle quality through mechanisms beyond proteolysis remains unclear.

*Chaenomeles speciosa* (*C. speciosa)* is an edible medicinal fruit with potential applications in food processing [6]. Its fruit contains various non-enzymatic bioactive constituents, particularly phenolic compounds, which have been reported to interact with proteins and influence their structural and functional properties [3,6,7,8]. In the present study, the total phenolic content and major phenolic compounds of the *C. speciosa* aqueous extract (SE) were characterized (Supplementary Materials Sections S2.1 and S2.2, and Table S2). These findings provide a basis for investigating the potential role of SE in regulating fish-muscle protein organization and processing quality. However, their effects in intact fish muscle and their distinction from acidity or proteolysis remain unclear, supporting further evaluation of SE as a natural tenderizing strategy that is not primarily dependent on proteolysis.

Rainbow trout is a commercially valuable fish species, and its processing quality is closely related to myofibrillar protein organization, water distribution, and texture characteristics [9]. Previous studies have shown that marination and thermal processing can modify fish-muscle structure, moisture characteristics, and flavor-related properties [10,11,12]. However, how plant-derived non-enzymatic constituents regulate fish-muscle protein networks during these processing conditions remains unclear. Consequently, achieving appropriate tenderization while maintaining favorable water-related and flavor properties represents an important practical challenge in rainbow trout processing.

Therefore, this work aimed to determine whether the SE could regulate rainbow trout muscle quality through mechanisms beyond acidification and proteolysis. By combining pH-matched controls, protease activity assays, and thermal inactivation experiments, we evaluated the effects of SE on texture, water retention, protein network organization, and flavor-related compounds under marination and boiling processing conditions. To further explore whether the effects of SE were associated with non-enzymatic bioactive compounds beyond protease activity, both marination and boiling conditions were applied in this study. Marination represented a relatively mild processing condition, whereas boiling provided a heat-treatment condition to evaluate whether the observed effects were maintained after protease inactivation. The loss of protease activity after boiling treatment was confirmed experimentally (Supplementary Section S3.1). This work provides insight into the potential role of heat-stable non-enzymatic constituents in regulating fish-muscle protein networks and highlights SE as a potential natural processing aid. By evaluating a whole aqueous extract through multiple quality and structural indicators, this work supports the potential use of SE as a natural, multifunctional complement to conventional protease-based tenderization. Further work on papain comparison, extract standardization, and practical application is needed.

## 2. Results and Discussion

### 2.1. Textural Properties and Shear Force

**Figure 1:**
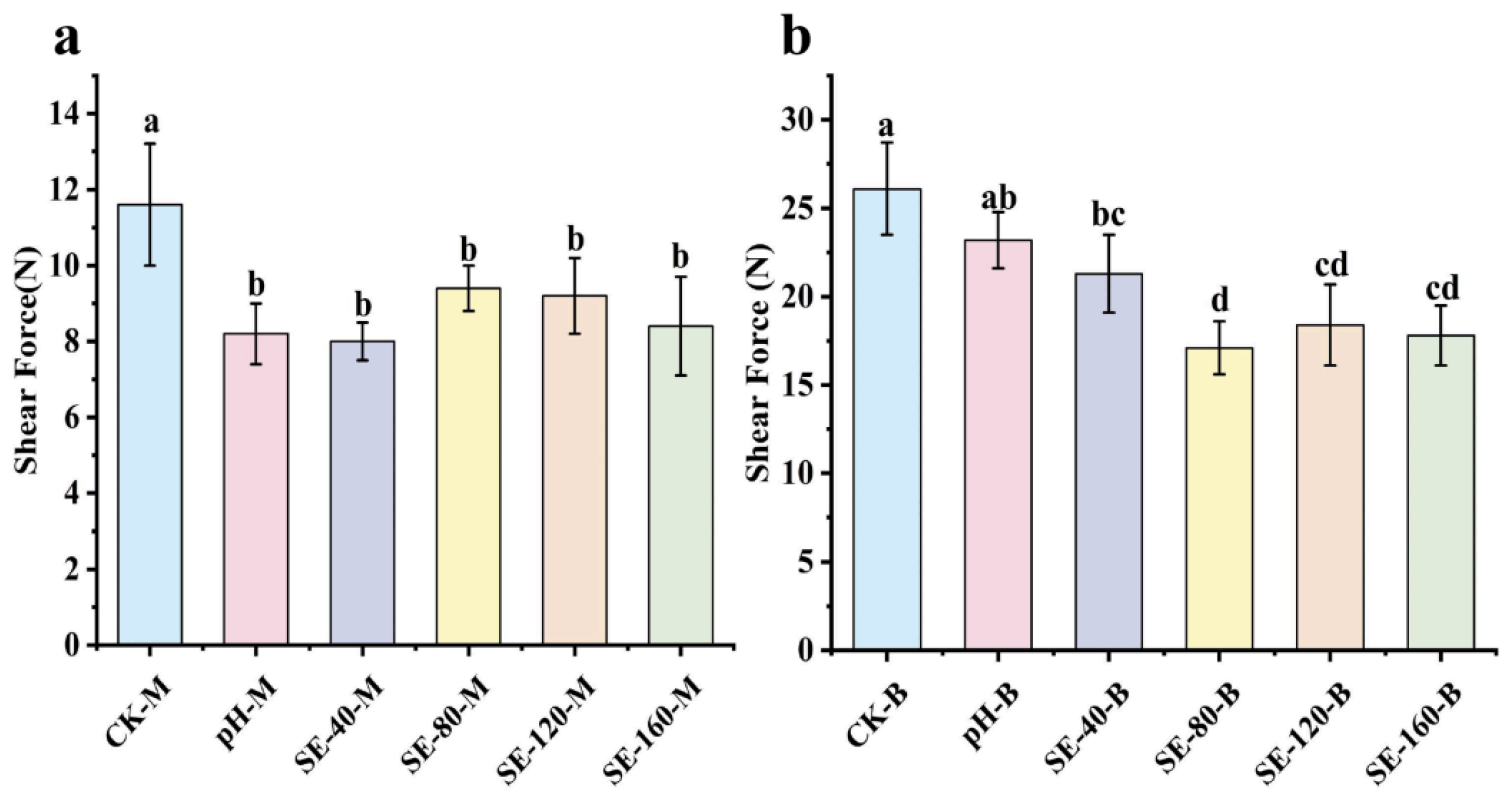
Shear force of rainbow trout myofibrillar networks treated with SE. (**a**) Marination treatment; (**b**) Boiling treatment. SE-40, SE-80, SE-120, and SE-160 represent SE concentrations of 40, 80, 120, and 160 mg/mL, respectively. M and B indicate marination and boiling treatments, respectively. Note: Data are presented as mean ± SD (*n* = 6). Different superscript letters indicate significant differences (Tukey’s HSD test, *p* < 0.05). Comparisons were performed within each processing condition.

The SE treatment was found to influence the textural properties of rainbow trout muscle under both processing conditions. As shown in Table 1 and Figure 1a, the hardness of the marinated samples decreased from 6.61 N in CK-M to 4.27–5.54 N in the SE-treated groups, while the shear force decreased from 11.60 N to 8.00–9.40 N. Compared with CK-M, SE treatment reduced shear force by approximately 19–31%. These changes indicate that SE softened the muscle tissue and reduced its resistance to cutting, possibly by modifying the organization of the myofibrillar network within the muscle structure.

A similar trend was observed in the boiling samples. As shown in Table 1 and Figure 1b, hardness decreased from 15.06 N in CK-B to 6.03–8.29 N in the SE-treated groups, while shear force decreased from 26.10 N to 17.80–21.30 N. Compared with CK-B, the reduction in shear force was approximately 18–32%. During boiling, heat-induced denaturation and aggregation of myofibrillar proteins promoted the formation and strengthening of a myofibrillar protein network within the muscle tissue, thereby increasing the baseline values of hardness and shear force [13]. Therefore, statistical comparisons were conducted within each processing condition using the corresponding control. The comparable relative reductions observed before and after boiling indicate that SE weakened or rearranged the myofibrillar protein network and that its texture-modifying effect was retained following thermal processing.

The SEM observations discussed below further support these texture results. After SE treatment, the myofibrillar structure became looser and more porous, accompanied by greater fiber disruption. These structural changes may have weakened the continuity and mechanical strength of the myofibrillar protein network, thereby contributing to the reductions in hardness and shear force. The more open microstructure may also have facilitated water retention; however, this interpretation should be considered together with the LF-NMR and water-distribution results.

These results are consistent with previous studies [14,15], which demonstrated that changes in the myofibrillar protein network and water distribution were closely associated with muscle texture. A less compact protein network and improved water retention may reduce the mechanical strength of muscle tissue, resulting in lower hardness and shear force. Overall, SE treatment modified the organization of the myofibrillar protein network and improved the texture of rainbow trout muscle under both processing conditions. However, the softening effect was not strictly concentration-related. Moderate SE concentrations showed more favorable effects, whereas further increases in SE concentration did not provide additional textural improvement. This suggests that SE regulates muscle structure through a balance between network relaxation and structural integrity preservation.

### 2.2. Water-Holding Capacity

**Figure 2:**
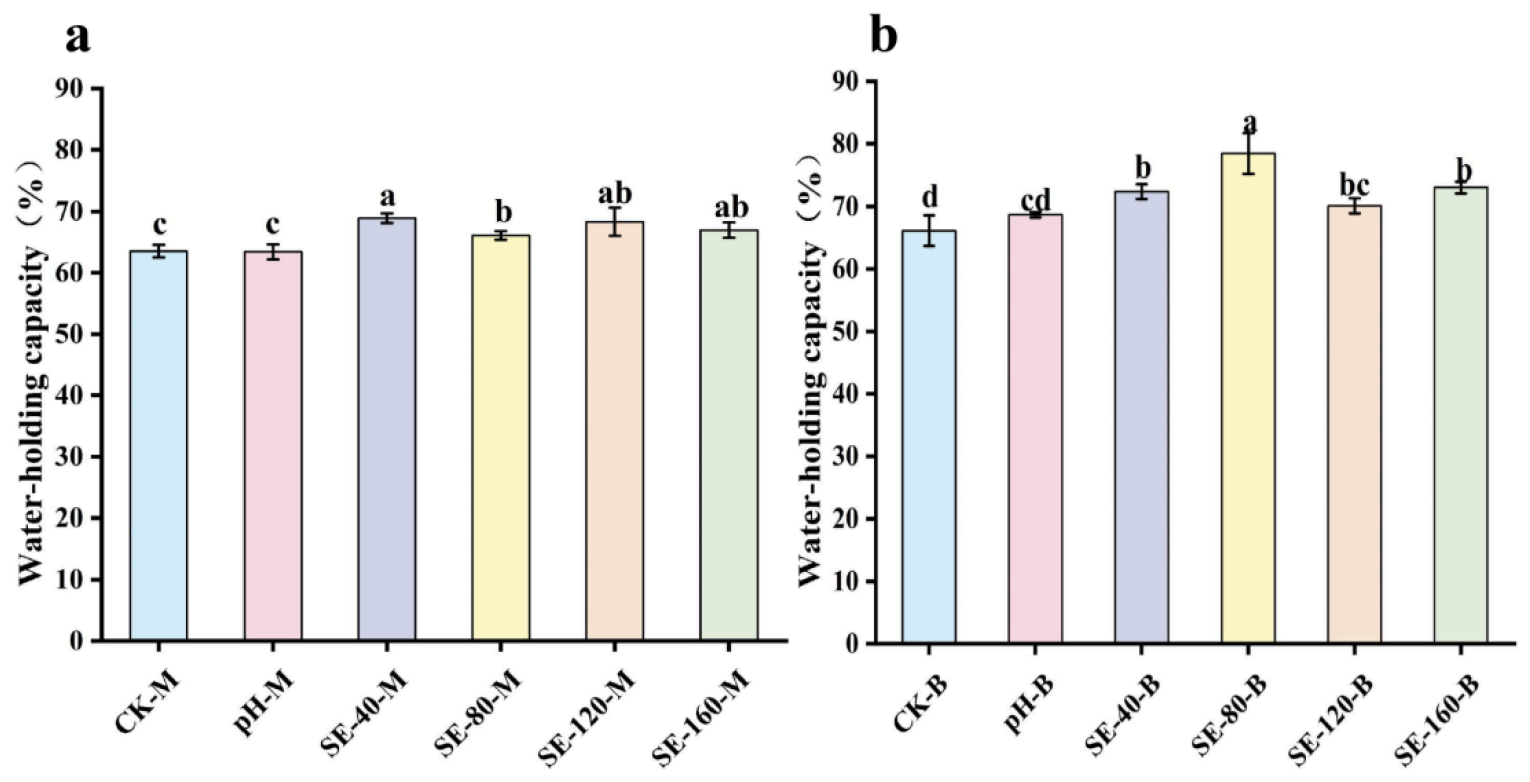
WHC of rainbow trout myofibrillar networks treated with SE. (**a**) Marination treatment; (**b**) Boiling treatment. SE-40, SE-80, SE-120, and SE-160 represent SE concentrations of 40, 80, 120, and 160 mg/mL, respectively. M and B indicate marination and boiling treatments, respectively.Data are presented as mean ± SD (*n* = 6). Different superscript letters indicate significant differences (Tukey’s HSD test, *p* < 0.05). Comparisons were performed within each processing condition.

As shown in Figure 2, SE treatment generally increased WHC values of rainbow trout muscle under both marination and boiling conditions, although the magnitude of the effect differed among SE concentrations and processing stages. In the marinated samples, all SE-treated groups exhibited higher WHC than CK-M and pH-M, but no clear concentration-related trend was observed. SE-40-M showed the highest WHC among the marinated samples, whereas further increases in SE concentration did not produce a consistent additional improvement. A more pronounced increase was observed after boiling. The WHC of SE-80-B reached approximately 78–79%, showing an improvement compared with CK-B and the pH-matched control. The increased WHC observed under boiling conditions may be associated with heat-induced protein unfolding, aggregation, and rearrangement, which promote the formation of a three-dimensional myofibrillar network. During boiling, myofibrillar proteins undergo unfolding, aggregation, and rearrangement, leading to the formation of a three-dimensional myofibrillar network capable of influencing water distribution through protein–water interactions and physical retention [16]. The highest WHC observed in SE-80-B suggests that this SE concentration may have facilitated interactions between SE components and muscle proteins, which could contribute to protein rearrangement and water retention. However, the reduction in WHC at higher SE concentrations indicates that the effect was not linearly concentration-related. Excessive interactions between polyphenolic compounds and proteins may promote network contraction or structural imbalance [17,18]. This rebound effect is important for practical application, because excessive SE addition may reduce water retention despite the beneficial effects observed at moderate concentrations. Therefore, optimizing the SE concentration is essential to achieve improved WHC without compromising protein network stability.

Previous works have indicated that plant extracts can delay the oxidation process in fish products and, under certain conditions, improve water retention properties. For example, research has shown that tea polyphenol soaking significantly improves the WHC of grass carp fillets, which is related to reduced oxidation and improved muscle protein structure [19]. Other studies have suggested that essential oil plant extracts, such as fennel essential oil, may enhance water retention in fish muscle tissue [1].

### 2.3. Protein Network Microstructure

**Figure 3:**
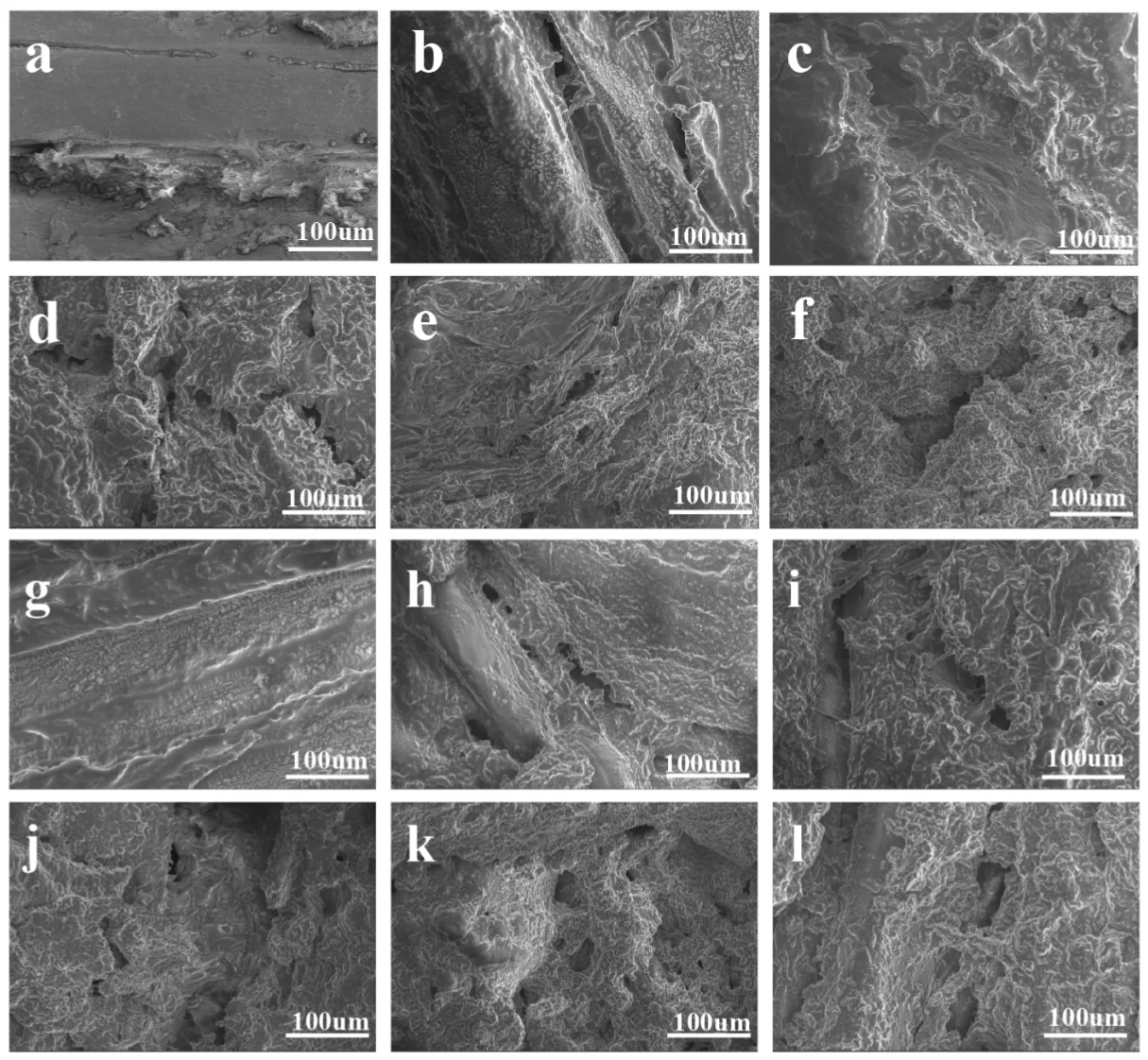
SEM images illustrating the effects of SE on the microstructure of rainbow trout myofibrillar networks. (**a**–**f**) Under marination (control and pH-matched control: SE-40-M to SE-160-M) and (**g**–**l**) under boiling conditions (corresponding groups).

The scanning electron microscopy (SEM) images clearly reveal the effects of SE on the tissue structure of rainbow trout muscle. Under marination conditions, as shown in Figure 3a–f, the control group (Figure 3a) exhibited well-ordered and dense muscle fibers, with minimal breakage or gaps. The pH-matched control (Figure 3b) exhibited relatively intact myofibrillar structures with limited visible separation between fibers, suggesting a minor effect of the acidic environment. In comparison, SE-treated groups (SE-40-M to SE-160-M, Figure 3c–f) showed concentration-dependent morphological alterations, including reduced compactness of myofibrils and visually increased separation between muscle fibers. These structural features appeared more evident at higher SE concentrations (120–160 mg/mL). These microstructural alterations correlate with the observed macroscopic tenderization. From a myofibrillar-network perspective, these changes suggest that SE altered the compact muscle protein matrix toward a looser and more porous myofibrillar network structure. Similar transitions from compact to loose/porous networks have been reported in heat- and additive-treated fish gels, which influence texture properties [20].

Similar patterns were observed under boiling conditions (Figure 3g–l). After thermal treatment, the control group (Figure 3g) and pH-matched control group (Figure 3h) maintained relatively continuous myofibrillar organization, suggesting limited structural disruption caused by heating alone or acidic conditions. In comparison, SE-treated groups (Figure 3i–l) exhibited visible changes in microstructural appearance, including reduced compactness of muscle structures and increased separation between muscle fibers. Notably, these tenderization effects persisted after thermal treatment, under which protease activity was undetectable, suggesting that heat-stable non-enzymatic bioactive components, including phenolic compounds identified in the extract, may contribute to the observed structural changes. This is consistent with previous studies [21], which observed that under lipid emulsification conditions, irregular network structures and pores/gaps appeared in the gel system as protein properties changed, and the fragmentation and looseness of these microstructures were similarly reflected in the SEM images.

Compared with both control groups, SE-treated samples exhibited differences in structural compactness and fiber arrangement, indicating that SE treatment altered the microstructural organization of the muscle protein matrix.

### 2.4. Water Distribution and Migration

**Figure 4:**
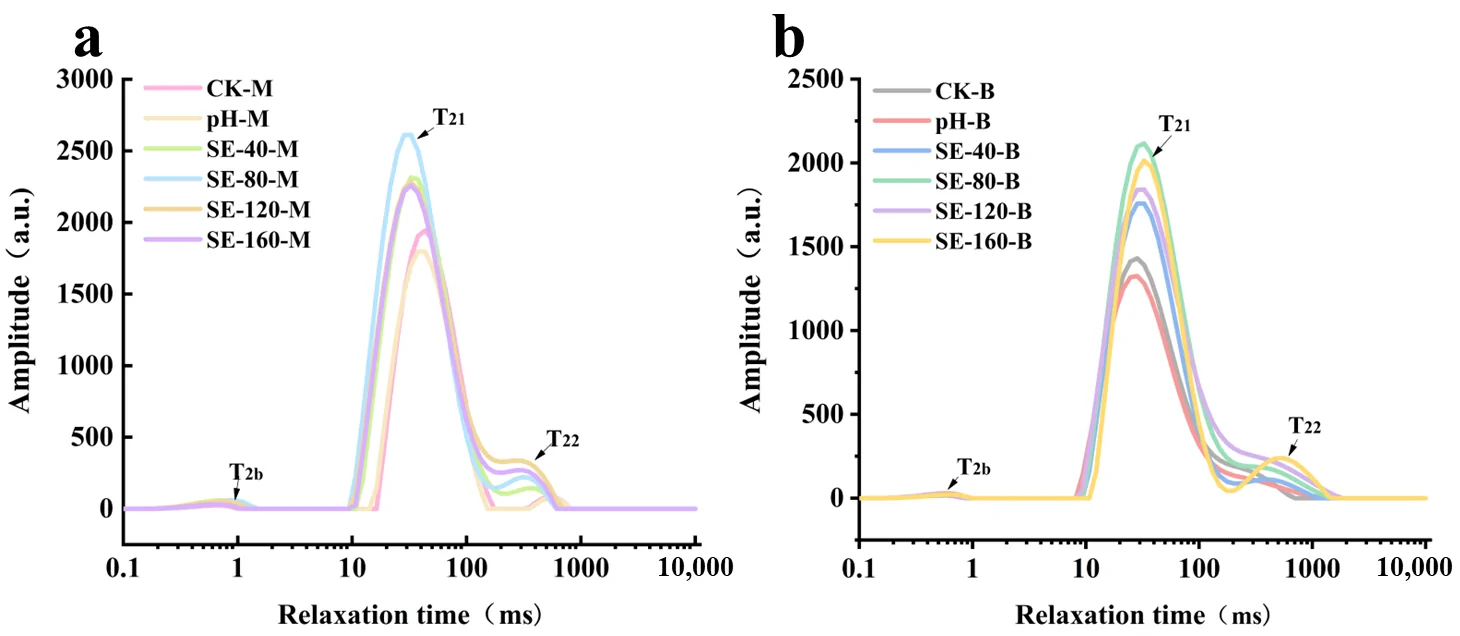
Changes in water relaxation times of rainbow trout myofibrillar networks treated with SE. (**a**) Marination treatment; (**b**) Boiling treatment. SE-40, SE-80, SE-120, and SE-160 represent SE concentrations of 40, 80, 120, and 160 mg/mL, respectively. M and B indicate marination and boiling treatments, respectively.

LF-NMR spectra of both marinated and boiled rainbow trout muscle showed three relaxation components, namely T_2b_, T_21_, and T_22_ (Figure 4). T_2b_ is generally associated with water tightly bound to protein macromolecules, T_21_ mainly reflects water with restricted mobility within the myofibrillar matrix, and T_22_ corresponds to a relatively more mobile water fraction. Among these components, T_21_ was the predominant relaxation signal in all samples. This difference in relaxation times helps assess the distribution and retention capacity of water within protein gels [10].

Under marination conditions (Figure 4a), SE treatment altered the distribution and relaxation profiles of T_2_ signals compared with the control and pH-matched control groups. Several SE-treated samples exhibited a shift in the T_21_ peak toward shorter relaxation times, suggesting a reduction in water mobility and an increased association between water molecules and muscle macromolecular structures. Meanwhile, variations in the T_22_ region were also observed, indicating that SE treatment influenced the relaxation behavior of the relatively less restricted water fraction. These results suggest that SE modified the distribution and molecular environment of water within rainbow trout muscle during marination.

After boiling (Figure 4b), the T_21_ peaks of different treatment groups became concentrated within a narrower relaxation-time range and showed increased overlap compared with those observed after marination. This indicates that thermal processing reduced the differences in water mobility among treatments, likely due to protein denaturation, structural rearrangement, and moisture redistribution during heating. Therefore, the effect of SE on water relaxation was more evident under marination conditions, whereas subsequent boiling partially attenuated the treatment-related differences in water distribution and mobility.

Overall, the LF-NMR results indicated that SE regulated water distribution in rainbow trout muscle, with more pronounced effects observed during marination. The shortened T_21_ relaxation times in several SE-treated samples suggested restricted water mobility within the myofibrillar matrix [22]. After boiling, these differences became less evident, likely due to the dominant influence of heat-induced protein restructuring.

### 2.5. Protein Surface Hydrophobicity

**Figure 5:**
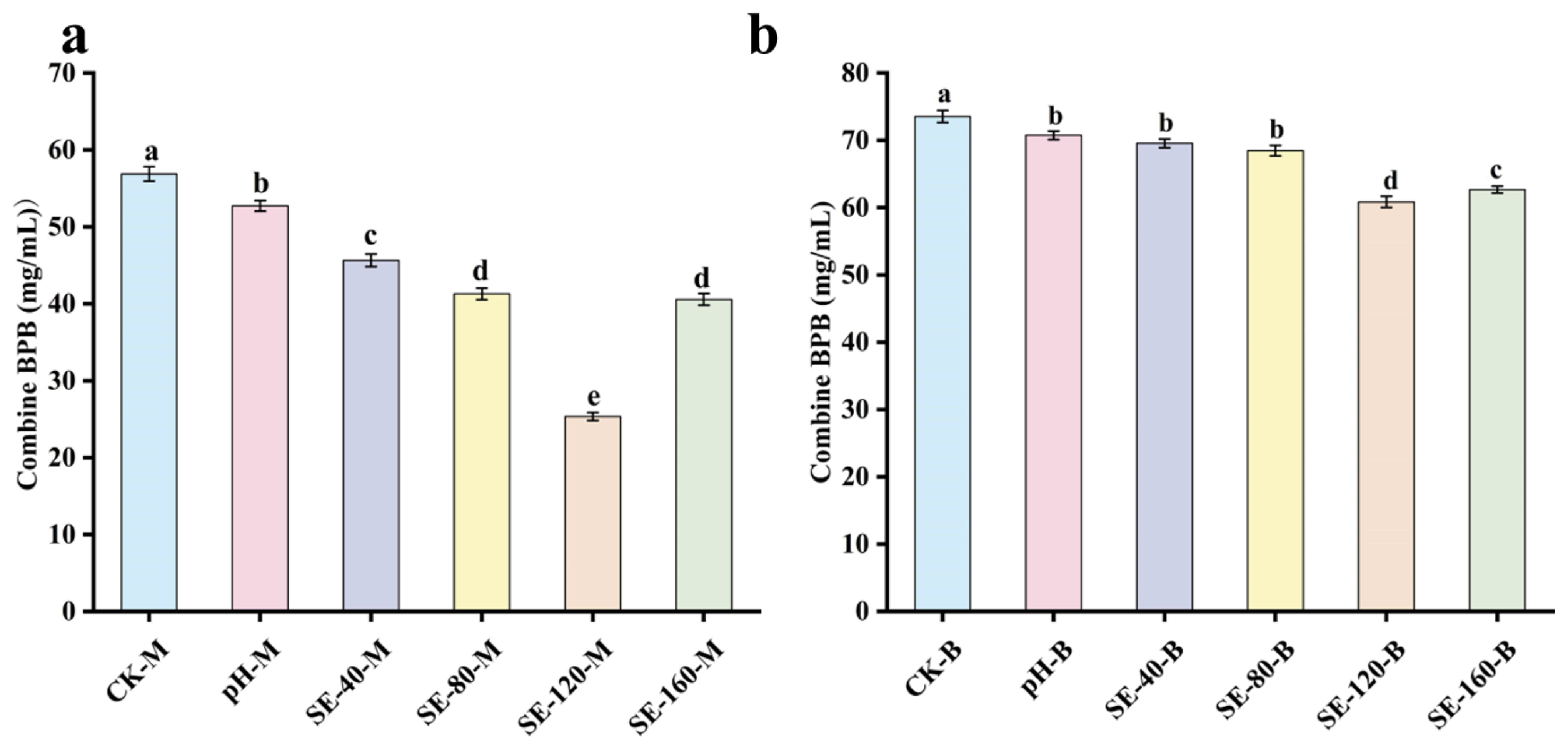
The effect of SE treatment on the surface hydrophobicity of rainbow trout muscle proteins. (**a**) Marination treatment; (**b**) Boiling treatment. SE-40, SE-80, SE-120, and SE-160 represent SE concentrations of 40, 80, 120, and 160 mg/mL, respectively. M and B indicate marination and boiling treatments, respectively. Note: Data are presented as mean ± SD (*n* = 6). Different superscript letters indicate significant differences (Tukey’s HSD test, *p* < 0.05). Comparisons were performed within each processing condition.

Protein surface hydrophobicity is an important parameter affecting the surface-related functions of proteins and serves as a key indicator reflecting the changes in the tertiary structure and aggregation behavior of myofibrillar proteins [23]. The results showed that under marination conditions (Figure 5a), SE treatment generally reduced the surface hydrophobicity of fish myofibrillar proteins, with a decreasing tendency observed with increasing SE concentration. Under boiling conditions (Figure 5b), moderate concentrations of SE partially alleviated the increase in hydrophobicity induced by thermal treatment.

This phenomenon suggests that the non-enzymatic bioactive compounds in SE may interact with the hydrophobic regions of proteins, leading to the reburial or shielding of some hydrophobic residues, thereby inhibiting heat-induced denaturation and hydrophobic aggregation. This effect is not merely a result of acidification but is more likely due to the stabilizing effect of small molecules, such as polyphenols, on the protein conformation [4]. At very high concentrations (160 mg/mL), a minor rebound in hydrophobicity was noted, possibly reflecting saturation or slight pro-oxidative effects. In food protein gels, surface hydrophobicity strongly affects protein aggregation and network formation. The decrease in surface hydrophobicity suggests that SE reduced excessive hydrophobic interactions, thereby preventing the formation of a compact and rigid myofibrillar network.

### 2.6. Thiol–Disulfide Bond Balance

**Figure 6:**
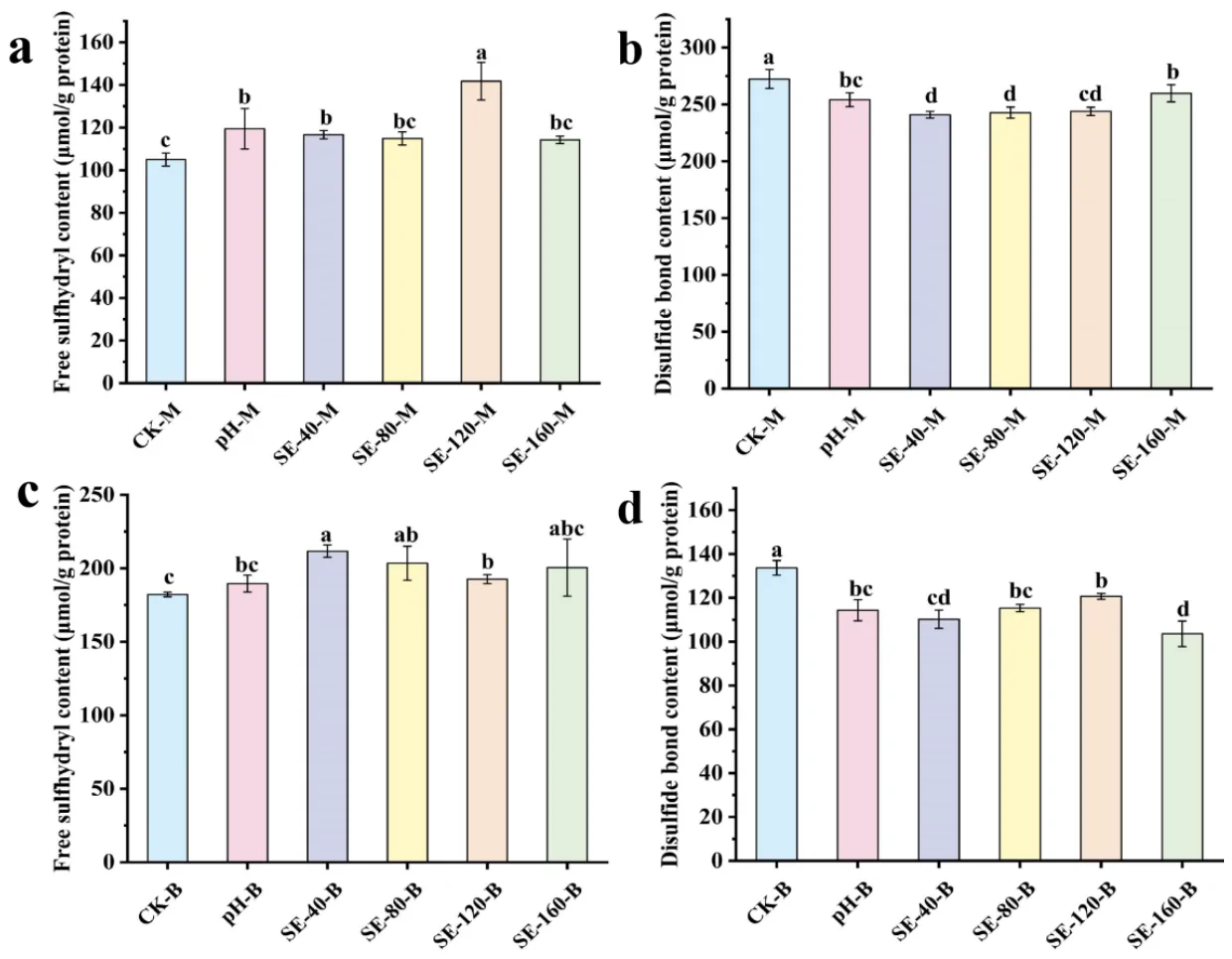
Effect of SE on free thiol groups and disulfide bonds in rainbow trout muscle proteins under two treatments. (**a**,**c**) Free sulfhydryl groups; (**b**,**d**) disulfide bonds. SE-40, SE-80, SE-120, and SE-160 represent concentrations of 40, 80, 120, and 160 mg/mL, respectively. M and B indicate marination and boiling treatments, respectively. Note: Data are presented as mean ± SD (*n* = 6). Different superscript letters indicate significant differences (Tukey’s HSD test, *p* < 0.05). Comparisons were performed within each processing condition.

Changes in thiol and disulfide bonds reflect the protein oxidation–crosslinking status. The results shown in Figure 6a indicate that SE treatment increased the free thiol content during marination, with significant increases observed mainly in the SE-40 and SE-80 groups compared with the control. This suggests that the non-enzymatic bioactive components may contribute to a mild conformational relaxation of the myofibrillar proteins, which could expose previously buried thiol groups while limiting further thiol oxidation and disulfide bond formation (Figure 6b). This finding is consistent with reports that polyphenolic compounds can interact non-covalently with proteins, inducing conformational changes that expose thiol groups while mitigating excessive oxidation [24,25].

In the boiling stage, as shown in Figure 6c,d, although heat treatment itself can promote thiol oxidation and disulfide bond exchange reactions [26], it also induces protein unfolding and sulfhydryl exposure, resulting in dynamic sulfhydryl/disulfide rearrangement rather than a simple conversion of thiols into disulfide bonds. Compared with the CK-B group, the SE-pretreated samples generally maintained higher free thiol contents and lower disulfide bond levels, although the magnitude of these changes varied among different SE concentrations. This indicates that the bioactive compounds in SE partially influenced the thiol–disulfide status and protein interaction behavior during thermal processing, thereby preserving a moderate crosslinking state within the myofibrillar network. This controlled structural modification likely facilitated subsequent protein rearrangement, leading to changes in protein network organization and protein–water interactions, which may contribute to the regulation of water retention properties.

These observations are supported by evidence that polyphenolic compounds can regulate protein structural stability and inhibit excessive oxidation under thermal or oxidative stress [24]. Therefore, the changes in thiol–disulfide status observed after SE treatment may reflect alterations in protein interactions and crosslinking behavior during thermal processing. SE appears to regulate rather than completely inhibit protein crosslinking, maintaining an appropriate balance between structural flexibility and network stability.

### 2.7. Protein Secondary Structure

**Figure 7:**
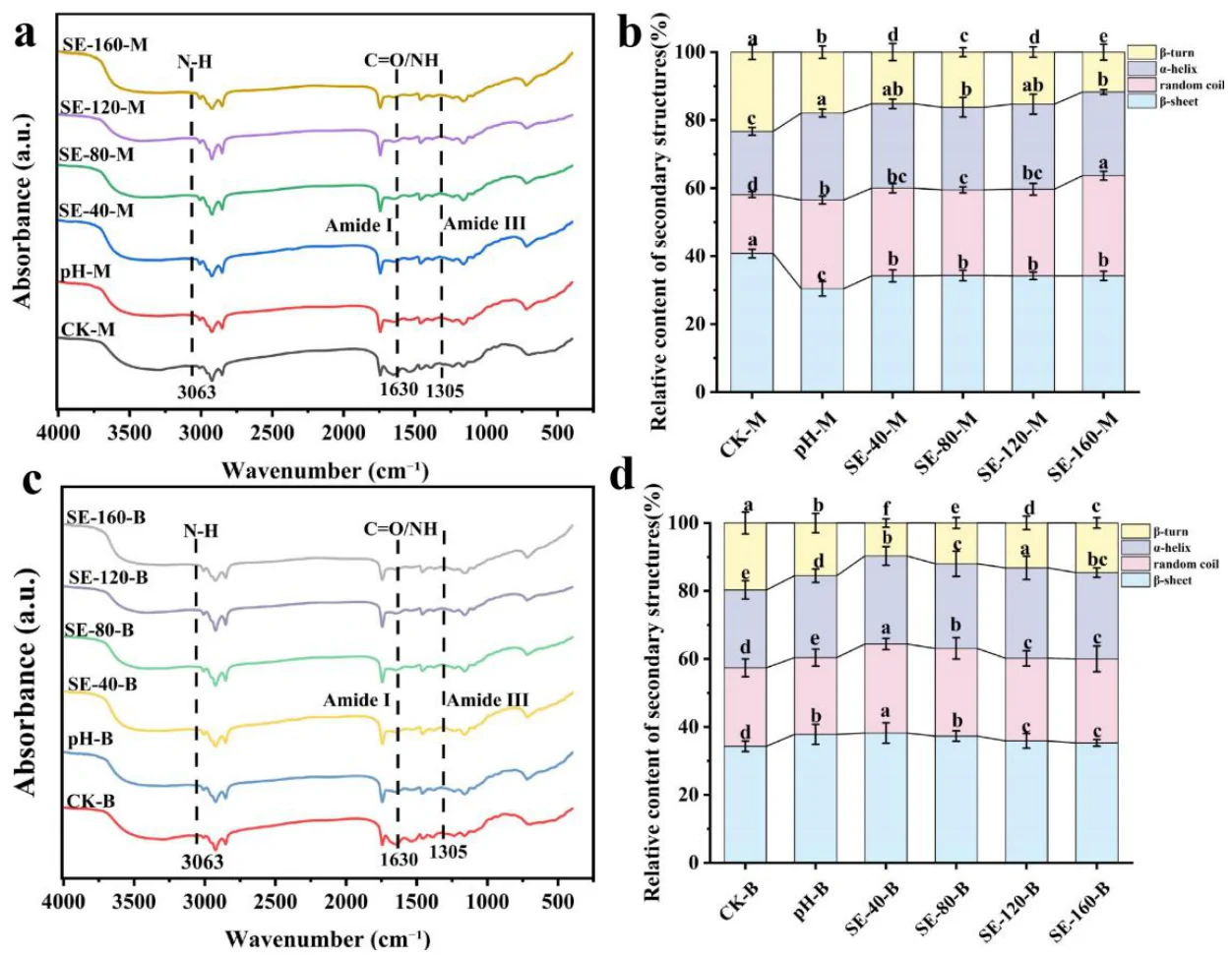
Effect of SE gradient on FTIR spectra (**a**,**c**) and changes in four secondary-structure components (**b**,**d**) of rainbow trout muscle proteins under two treatments. SE-40, SE-80, SE-120, and SE-160 represent concentrations of 40, 80, 120, and 160 mg/mL, respectively. M and B indicate marination and boiling treatments, respectively. Note: Data are presented as mean ± SD (*n* = 6). Different superscript letters indicate significant differences (Tukey’s HSD test, *p* < 0.05). Comparisons were performed within each processing condition.

In the marination group, as shown in Figure 7a,b, treatment with SE altered the secondary structure composition of myofibrillar proteins, with decreases in β-sheet and β-turn contents and increases in random coil and α-helix contents observed in several SE-treated groups. These changes suggest that SE induces a shift from β-sheet to α-helix and random coil structures. β-sheet is typically associated with tight aggregation and rigidity in proteins. SE may increase structural flexibility (random coil) and, combined with reduced surface hydrophobicity, leads to a decrease in hardness and shear force of the protein network at the macroscopic level (tenderization effect), while exhibiting a porous and elastic network at the microscopic level. Similar secondary structure changes have been reported in previous studies [27], indicating that under marination and non-thermal conditions, protein structures become more relaxed and flexible. At the same time, as mentioned earlier, the slight decrease in disulfide bond levels indicates that the relaxation of protein conformation leads to the exposure of previously buried thiol groups, without excessive oxidation or crosslinking. The changes in secondary structure and thiol exposure together reflected conformational rearrangement of proteins, which may contribute to the flexibility of the myofibrillar network. These secondary-structure changes provide a molecular basis for the formation of a softer and more flexible myofibrillar network.

In the boiling group (Figure 7c,d), changes in β-sheet content were relatively minor compared to the control, while random coil content increased and β-turn content decreased in SE-treated samples. The relatively limited variation in β-sheet content may be mainly attributed to the dominant influence of boiling on protein conformational changes. Compared with marination without heating, boiling induced extensive protein unfolding and aggregation in all treatment groups, which may have reduced the relative contribution of SE-induced conformational modulation.

However, considering that boiling at 95 °C is the dominant factor driving protein unfolding and aggregation, the secondary structure variations among SE-treated groups should be interpreted as subtle conformational adjustments rather than substantial reversal of heat-induced structural changes. The relatively limited variation in β-sheet content, together with changes in random coil and β-turn structures, suggests that SE may influence heat-induced conformational rearrangement rather than completely prevent thermal denaturation. This interpretation is consistent with a previous study [28], which reported an increase in random coil content and a decrease in β-turn content during high-temperature treatment, reflecting protein conformational adjustment associated with gel formation. The observed changes in disulfide-bond content may reflect alterations in protein interactions during heat-induced rearrangement rather than a direct indication of reduced cross-linking. These results, together with the changes in protein surface hydrophobicity, thiol–disulfide status, and FTIR analysis, suggest that SE modulated the conformational reorganization of myofibrillar proteins during thermal processing. Overall, SE may influence heat-induced protein rearrangement through multiple structural interactions, including changes in conformation, intermolecular interactions, and network organization.

### 2.8. Fishy Odor-Related Compounds

GC-MS analysis results shown in Table 2 indicate that 23 volatile compounds were identified in rainbow trout, including 4 alcohols, 13 aldehydes (5 saturated fatty aldehydes, 7 unsaturated fatty aldehydes, and 1 aromatic aldehyde), 1 ketone, and 5 hydrocarbons. The analysis indicated that hexanal, heptanal, (E)-2-heptenal, 1-octen-3-ol, octanal, and nonanal are the primary compounds responsible for the fishy odor. The effects of different treatment conditions on these compounds were analyzed. SE treatment affected the distribution of short-chain (C6–C7) and long-chain aldehydes (C8–C9). Hexanal, heptanal, (E)-2-heptenal, as well as octanal and nonanal, are commonly regarded as typical lipid oxidation products and key contributors to the fishy/oxidized odor in fish and fish products [29]. Among these, hexanal, nonanal, and 1-octen-3-ol are confirmed as major fishy odor markers in various aquatic products [30].

In the marination group, as shown in Table 3, compared to the CK-M group, treatment with the SE significantly reduced the content of C6–C7 short-chain aldehydes, especially hexanal, which decreased by nearly one-third in the SE-40-M group. (E)-2-heptenal also showed a decreasing trend in all treatment groups. Long-chain aldehydes, such as octanal and nonanal, were lower in all SE-treated groups than in the CK-M group, with a moderate reduction. This suggests that SE treatment during marination tends to weaken short-chain aldehydes associated with “grassy/raw fish” odors and early lipid oxidation, while also inhibiting the formation of long-chain aldehydes like octanal and nonanal, thus reducing the overall fishy potential of the fish meat system before entering the heating process.

In the boiling group, as shown in Table 3, (E)-2-heptenal and 1-octen-3-ol were below the detection limit in all boiled samples, indicating that thermal processing itself may be the major factor influencing the changes in these volatile compounds. The reduction or disappearance of these compounds after boiling may be related to volatilization losses and heat-induced degradation or transformation during thermal processing, which is consistent with the reported pathways of polyunsaturated fatty acid (PUFA) degradation during heating [31]. Compared to CK-B, low-to-moderate intensity SE treatments (pH-B, SE-40-B) significantly reduced the total content of hexanal and heptanal, while continuously decreasing the content of octanal and nonanal in all boiling groups. These results suggest that SE may modulate the formation of specific lipid oxidation-related volatile compounds during thermal processing. However, under the high-intensity treatment condition (SE-160-B), hexanal content increased again and exceeded that of CK-B, suggesting that excessive SE addition may alter the balance between antioxidant and pro-oxidant effects. Although moderate levels of plant polyphenols are generally effective in retarding lipid oxidation through radical scavenging and metal chelation, excessively high concentrations may promote polyphenol autoxidation, leading to the formation of reactive oxygen species and quinone-type intermediates with potential pro-oxidant activity [32]. Meanwhile, the pronounced disruption of muscle microstructure under SE-160-B may have increased lipid accessibility to oxygen, thereby facilitating oxidative reactions. Notably, the effects of SE on volatile compounds were concentration-related. While low-to-moderate SE concentrations reduced oxidation-related volatile compounds, the increased hexanal content in the SE-160-B group suggested a rebound effect under excessive treatment conditions. This may result from enhanced lipid exposure and possible pro-oxidant effects of phenolic compounds at high concentrations. Therefore, optimizing the SE concentration is essential to balance flavor improvement and oxidative stability. From the perspective of protein structure–flavor interactions, the modulation of volatile compounds by SE may not be attributed solely to the inhibition of lipid oxidation, but may also be associated with changes in the binding and release behavior of odor-active compounds induced by conformational alterations in myofibrillar proteins. A previous work [33] investigated a model interaction system consisting of grass carp myofibrillar proteins and standard fishy compounds, and showed that myofibrillar proteins exhibited different binding capacities toward fishy odorants depending on compound type and chain length. Although the model system differed from the sample matrix used in the present study, these findings may provide useful insights for interpreting the changes in volatile compounds observed in our work. It is reasonable to infer that SE-induced structural alterations of myofibrillar proteins may have influenced the odor-binding sites of proteins, thereby affecting the retention and release behavior of key volatile compounds. Thus, the reduction in aldehydes and 1-octen-3-ol in most SE-treated groups may reflect not only suppressed lipid oxidation but also possible changes in volatile–protein interactions. Under excessive treatment intensity, however, more pronounced alterations of the muscle structure may have increased lipid exposure during heating, which may have contributed to the rebound in hexanal content after boiling.

## 3. Conclusions

This work demonstrates that SE can improve the functional properties of rainbow trout muscle by regulating the organization and rearrangement of myofibrillar networks during marination and heating. SE improved texture, water retention, and odor-related characteristics, which may be associated with changes in protein conformation, crosslinking behavior, water immobilization, and microstructural organization. Rather than causing isolated quality changes, SE reshaped protein physicochemical transitions during marination and heating, ultimately promoting a more favorable structural and sensory outcome. More importantly, the additional effects of SE beyond acidification may be associated with its heat-stable non-enzymatic constituents, including phenolic compounds identified in the extract. Unlike conventional pH adjustment or enzymatic softening, SE may modulate protein unfolding, intermolecular interactions, and aggregation behavior during processing, resulting in differences in the organization and compactness of the post-heating myofibrillar protein network. Such structural rearrangement may contribute to the formation of a more balanced protein network with improved water retention characteristics. These findings suggest that SE has potential as a natural processing aid for cold-water fish products, especially rainbow trout, by improving texture and odor without synthetic additives.

However, its practical application still requires further study, particularly with respect to treatment optimization, consistency under different processing conditions, and feasibility for industrial-scale use. Future work should therefore focus on further refining application parameters, identifying key factors influencing SE functionality, and validating its effectiveness and stability under real processing and storage conditions. Moreover, we should evaluate the reproducibility and robustness of SE bioactivity across independent extraction batches and establish standardized extraction protocols to ensure consistent quality for practical applications. Overall, this work provides a gel-science-based interpretation suggesting that plant-derived non-enzymatic bioactive compounds may serve as a potential approach for modulating fish muscle protein networks, supporting the potential application of SE in the development of high-quality cold-water fish products.

## 4. Materials and Methods

### 4.1. Materials and Reagents

Six fresh rainbow trout of similar size (approximately 1.5–2.0 kg body weight and 40–50 cm body length) were purchased from the Kunming Aquatic Market (Kunming, Yunnan, China) and used as six independent biological sources. After slaughter, the fish were skinned and deboned, and dorsal muscle from the same anatomical region was collected and cut into blocks measuring 20 mm × 10 mm × 10 mm. Muscle blocks derived from each individual fish were randomly allocated to all treatment groups, ensuring that each treatment condition included samples originating from all six individual fish. Fresh *C. speciosa* fruits were harvested from Lincang (Yunnan, China) and selected for uniform ripeness and the absence of mechanical damage. All chemical reagents were of analytical grade and were purchased from Shanghai Aladdin Biochemical Technology Co., Ltd. (Shanghai, China).

### 4.2. Preparation and Treatment of SE

*C. speciosa* was thoroughly washed and sliced according to previously described methods with slight modifications [34,35]. Three separately weighed portions from the same batch of *C. speciosa* were used to prepare three independent extraction replicates. For each replicate, the slices were homogenized in deionized water at a solid-to-liquid ratio of 1:10 (*w*/*v*), followed by water-bath extraction at 60 °C for 30 min and ultrasonication at 30 kHz using an ultrasonic processor (XiaoMei Ultrasonic Instruments, Kunshan, China). Each extract was then centrifuged at 8000× *g* for 15 min using a refrigerated high-speed centrifuge (Multifuge X1 R, Thermo Fisher Scientific, Waltham, MA, USA), and the supernatant was collected separately. The three independently prepared aqueous extracts were freeze-dried separately under identical conditions within the same freeze-drying run using a vacuum freeze dryer (SCIENTZ-12 N/C, Ningbo Scientz Biotech Co., Ltd., Ningbo, China) to minimize processing variability and ensure consistency in subsequent experiments. Each freeze-dried extract was subsequently re-dissolved in deionized water to obtain concentrations of 40, 80, 120, and 160 mg/mL for subsequent experiments.

### 4.3. Sample Treatment Methods

Marination and boiling treatments were included primarily to distinguish protease-dependent effects from those associated with the non-enzymatic constituents of SE. Marination at 4 °C represented a condition under which the low protease activity detected in SE remained active and could potentially contribute to changes in rainbow trout muscle. In contrast, boiling at 95 °C completely inactivated the protease activity of SE, as experimentally verified in Supplementary Section S3.1. Therefore, marination and boiling treatments were used to evaluate the effects of SE on rainbow trout muscle protein networks under conditions in which protease activity was retained or completely inactivated by heat, respectively.

**Figure 8:**
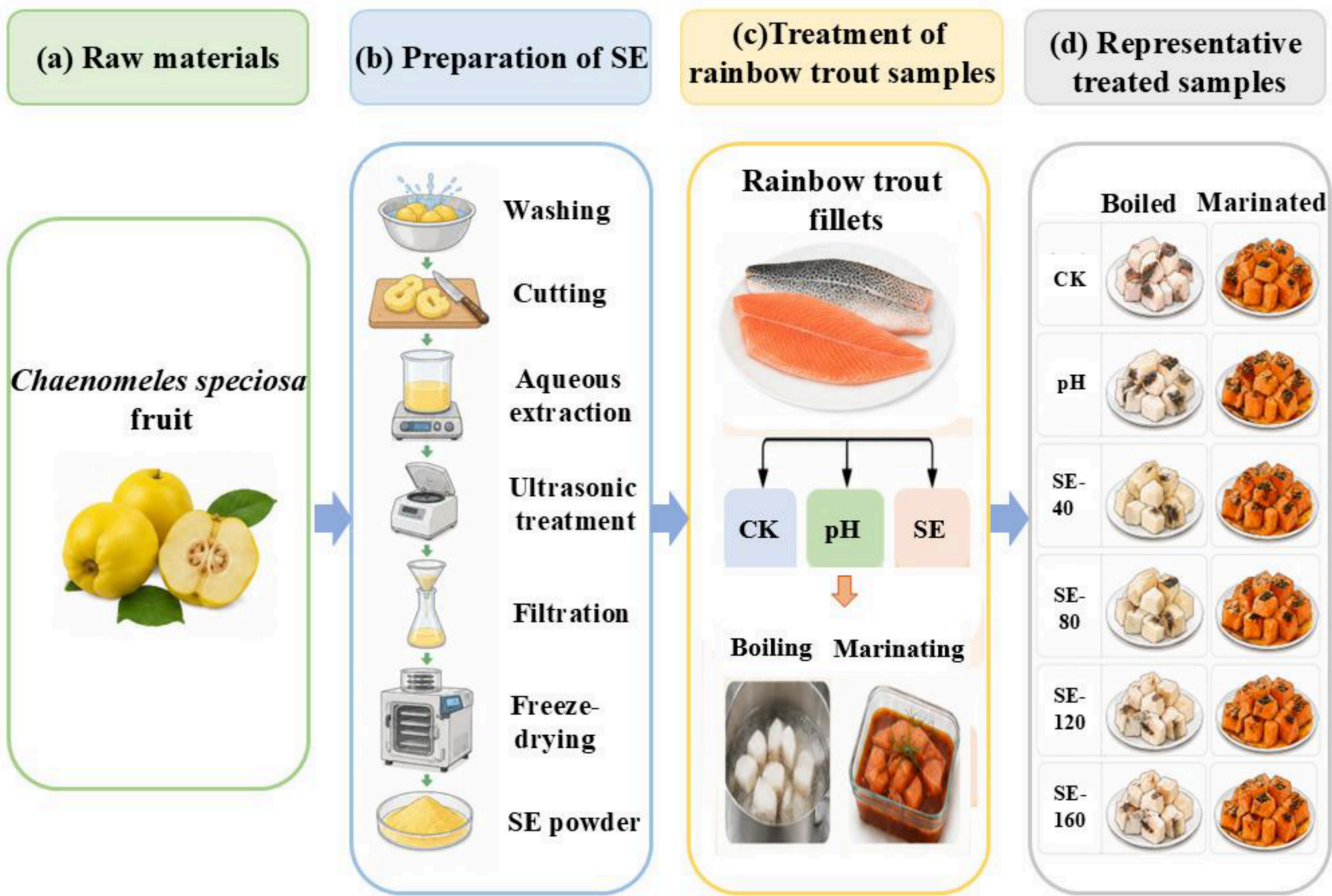
Schematic illustration of raw materials, preparation of *C. speciosa* aqueous extract (SE), and treatment of rainbow trout samples. Samples were treated with distilled water (CK, blank control), pH-matched malic acid solution (pH 2.76), or SE at 40, 80, 120, and 160 mg/mL (SE-40–SE-160).

The dorsal muscle blocks prepared as described in Section 4.1 were immersed in the corresponding treatment solutions at a sample-to-liquid ratio of 1:2 (*w*/*v*). Samples assigned to the marination groups (M) were treated at 4 °C for 30 min. Samples assigned to the boiling groups (B) were heated at 95 °C for 3 min, followed by cooling and equilibration at 4 °C for 30 min in the same solutions. For each treatment, samples were immersed in distilled water (blank control, CK), malic acid solution adjusted to match the pH of SE (pH-matched control, pH = 2.76; see Supplementary Section S2.1), or SE at 40, 80, 120, and 160 mg/mL (SE-40, SE-80, SE-120, and SE-160, respectively). Group labels reflected both the treatment condition and SE concentration (e.g., SE-40-M for marinated samples and SE-40-B for boiled samples). This experimental design allowed comparison of the effects of SE under different processing conditions. A schematic overview of the raw materials, preparation of the SE, and treatment procedure for the rainbow trout samples is presented in Figure 8.

### 4.4. Measurement of Protein Surface Hydrophobicity

All analytical samples were prepared from muscle blocks obtained from the six individual rainbow trout described in Section 4.1. Samples from different fish were included in each treatment to incorporate biological variation. Unless otherwise specified, the same sample preparation procedure was used for subsequent analyses.

Protein surface hydrophobicity can be assessed by measuring the binding affinity of the hydrophobic chromophore bromophenol blue (BPB). The assay was performed following a previously reported method with slight modifications [36]: 2 g muscle subsamples derived from the prepared muscle blocks were mixed with 10 volumes of Phosphate-buffered saline (PBS) (20 mmol/L, pH 6) and homogenized at 18,000 rpm using a high-speed disperser (T 25 digital, IKA-Werke GmbH & Co. KG, Staufen, Germany). The protein concentration of the homogenate was determined by the Biuret method and adjusted to 5 mg/mL. Subsequently, 1 mL of the sample suspension or PBS (control) was mixed with 200 μL of BPB solution (1 mg/mL) by vortexing. After stirring at room temperature for a specified time, the mixtures were centrifuged at 4 °C and 2000× *g* for 15 min. The supernatant was collected, diluted 10-fold with PBS, and the absorbance at 595 nm was measured using a microplate reader (EPOCG2, BioTek Instruments, Inc., Winooski, VT, USA). The BPB binding capacity was calculated using Equation (1):

(1) $$BPB\;binding=200\;ug\times \frac{Ac-As}{Ac}$$

where *Ac* and *As* represent the absorbance values of the control and sample at 595 nm, respectively.

### 4.5. Measurement of Thiol and Disulfide Bond Content

#### 4.5.1. Determination of Total Thiol (–SH) Content

Total thiol (–SH) content was determined according to a previously reported method [36]: a 4 g muscle subsample derived from the prepared muscle blocks was dispersed in 40 mL of 0.2 mol/L Tris-HCl buffer (pH 8.0) containing 3 mmol/L EDTA, 8 mol/L urea, and 2% SDS, and homogenized at 12,000 rpm for 1 min using a high-speed disperser. The homogenate was heated for 2 min in boiling water and stirred at room temperature for 4 h. After centrifugation (4 °C, 10,000× *g*, 15 min), the supernatant was collected and adjusted to 1 mg/mL protein. Then, 4 mL of the supernatant was mixed with 0.4 mL Ellman’s reagent, incubated at 40 °C for 15 min, and absorbance was measured at 412 nm. The –SH content was calculated using Equation (2) and expressed as μmol/g protein:

(2) $$-SH\;content=\frac{A_{412}\times D\times 10^{6}}{C\times 13,600}$$

where *A*_412_ is the absorbance at 412 nm; *D* is the dilution factor of the supernatant; *C* is the protein concentration in the supernatant (mg/mL); and 13,600 is the molar extinction coefficient (M^−1^ cm^−1^).

#### 4.5.2. Free Thiol and Disulfide Bond Content

The free thiol content was determined according to a previously reported method [37]. Briefly, protein solutions prepared from the treated muscle samples were adjusted to a concentration of 1 mg/mL. Then, 5.5 mL of the prepared protein solution was mixed with 100 μL of DTNB (10 mmol/L) and allowed to react for 15 min. The absorbance of the mixture was measured at a specific wavelength of 412 nm. Free and total thiol contents were calculated using the equations described above and expressed as μmol/g protein. The disulfide bond content was calculated using the following Formula (3):

(3) $$Disulfide\;bond=\frac{Total\;thiol\;-\;Free\;thiol}{2}$$

### 4.6. Measurement of Protein Secondary Structure

The protein secondary structure was carried out according to a previously reported method [36]. Lyophilized samples were mixed with dried potassium bromide (1:100, *w*/*w*) and pressed into pellets. Fourier transform infrared spectroscopy (FTIR) was obtained using a VERTEX 70 spectrometer (Bruker Optics GmbH & Co. KG, Ettlingen, Germany) over 4000–400 cm^−1^ at a resolution of 4 cm^−1^. The amide I region (1600–1700 cm^−1^) was used for secondary structure analysis, and peak fitting was performed using Peakfit softwareversion 4.12 (Systat Software Inc., San Jose, CA, USA).

### 4.7. Observation of the Ultrastructural Organization of Rainbow Trout

The ultrastructural organization of rainbow trout muscle was observed according to a previously reported method [36]. Thin sections of the prepared muscle blocks, approximately 1 mm thick, were placed in 2.5% glutaraldehyde (pH 7.0) containing 0.1 mol/L PBS and fixed at 4 °C for 24 h. The samples were then washed three times with 0.1 mol/L PBS, each wash lasting 10 min. Subsequently, the samples were dehydrated with ethanol solutions of 30%, 50%, 70%, 80%, 90%, and 100%, with each solution applied for at least 10 min. After freeze-drying, the ultrastructure of the fish muscle tissue was observed using a scanning electron microscope (Quattro S, Thermo Fisher Scientific, Hillsboro, OR, USA). The accelerating voltage was set at 3.0 kV, and the magnification was 1000×.

### 4.8. Low-Field Nuclear Magnetic Resonance (LF-NMR) Measurement

The LF-NMR was carried out according to a previously reported method [36]. Dorsal muscle was collected and cut into blocks measuring 20 mm × 10 mm × 10 mm, and then wrapped in cling film and placed in an LF-NMR analyzer (NMI20, Shanghai NMI Electronic Technology Co., Ltd., Shanghai, China) to determine the moisture content. The sampling bandwidth was set to 100 kHz, with an echo time of 0.8 s, 5000 echoes, a delay time of 5000 ms, and 2 accumulations. The analysis focused on the bound water, immobile water, and the changes in free water under different treatment methods.

### 4.9. Texture and Shear Force Measurement

Texture properties were measured according to a previously reported method [36]. Dorsal muscle was collected and cut into blocks measuring 20 mm × 10 mm × 10 mm. At room temperature, texture measurements were performed using a texture analyzer (TA. XT plus, Stable Micro Systems Ltd., Godalming, Surrey, UK) equipped with a P/36R probe. The test was conducted at a speed of 1.0 mm/s, with a trigger force of 5 g, a compression interval of 5 s, and a compression rate of 70%. The texture parameters, including hardness, chewiness, elasticity, and cohesiveness, were calculated from the force–deformation curve.

For shear force measurement, dorsal muscle was collected and cut into blocks measuring 20 mm × 10 mm × 10 mm. The shear force of the fish blocks was measured using a physical property analyzer equipped with an HDP/BS crosshead. The main parameters were set as follows: pre-test, middle, and post-test speeds were 2, 2, and 10 mm/s, respectively, with a trigger force of 5 g and a downward distance of 30 mm.

### 4.10. Water Holding Capacity (WHC) Measurement

The WHC was performed following a previously published method [38]; the difference in water retention capacity was calculated by determining the centrifugal loss of the sample. The specific method is as follows: The muscle subsamples derived from the prepared muscle blocks were cut into pieces weighing 5 g (*W*_0_), and excess surface moisture was carefully removed using absorbent paper. The sample was subsequently enclosed in filter paper, placed into a 50 mL centrifuge tube, and centrifuged at 4 °C and 1000× *g* for 10 min. After centrifugation, any remaining surface water was eliminated, and the sample was reweighed (*W*_1_). Centrifugal loss was determined based on Equation (4):

(4) $$Water\;holding\;capacity=\frac{W_{0}-W_{1}}{W_{0}}\times 100\%$$

where *W*_0_ is the weight of the meat before centrifugation, and *W*_1_ is the weight of the meat after centrifugation.

### 4.11. Determination of Fishy Odor Compounds in Rainbow Trout

#### 4.11.1. Sample Preparation

Following the treatments described in Section 4.3, dorsal muscle samples derived from the prepared biological sources were separately minced for each treatment group and immediately subjected to volatile compound analysis.

#### 4.11.2. Headspace Solid-Phase Microextraction (HS-SPME)

HS-SPME analysis was performed according to a previously reported method [39]. Volatile compounds in minced muscle subsamples were extracted using the HS-SPME method. Briefly, approximately 5 g of minced muscle subsample and 6 μL of 2-methyl-3-heptanone solution (0.82 μg/mL, as the internal standard) were transferred into a headspace vial. The mixture was first equilibrated in a water bath at 60 °C for 20 min. Volatile components were then adsorbed for 40 min at 60 °C with an HS-SPME fiber needle (50/30 μm, divinylbenzene/carboxen/polydimethylsiloxane fiber, Supelco, Bellefonte, PA, USA). Once extraction was completed, the fiber was promptly introduced into the GC-MS system for analysis, and the analytes were thermally desorbed at 250 °C for 5 min.

#### 4.11.3. Gas Chromatography–Mass Spectrometry (GC-MS) Analysis

Volatile odor-active compounds were analyzed by GC–MS. Separation was achieved using a DB-5 ms capillary column (30 m × 0.32 mm, 0.25 μm film thickness; J&W Scientific, Folsom, CA, USA). The oven temperature was initially set at 40 °C and held for 3 min, and then increased to 230 °C at a rate of 4 °C/min and held at this temperature for another 3 min. Ultra-high-purity helium (99.999%, Beijing AP Baifu Gas Industrial Co., Ltd., Beijing, China) served as the carrier gas. Electron impact ionization was operated at 70 eV, with the mass scanning range set from *m*/*z* 25 to 370 amu. The ion source and quadrupole temperatures were set at 230 °C and 150 °C, respectively.

### 4.12. Statistical Analysis

Data were analyzed by one-way analysis of variance (ANOVA) using SPSS Statistics 27 software (SPSS Inc., Chicago, IL, USA), and significant differences among means were determined by Tukey’s HSD test at *p* < 0.05. For quantitative analyses, biological replicates were derived from six individual fish for each treatment condition. Technical repetitions were performed where necessary according to the requirements of each analytical method, and averaged values were used for statistical analysis. Data were plotted using Origin Pro 2021 software (Origin Lab Corporation, Northampton, MA, USA).

## Figures and Tables

**Table 1:** Changes in Texture Profile Parameters of Rainbow Trout Muscle under SE Treatment. SE-40, SE-80, SE-120, and SE-160 represent concentrations of 40, 80, 120, and 160 mg/mL, respectively. M and B indicate marination and boiling treatments, respectively.

Treatment	Hardness (N)	Elasticity	Cohesiveness	Chewiness	Resilience
CK-M	6.61 ± 0.39 ^ab^	0.27 ± 0.05 ^a^	0.55 ± 0.11 ^ab^	100.69 ± 8.73 ^a^	0.18 ± 0.02 ^ab^
pH-M	7.72 ± 1.73 ^a^	0.23 ± 0.05 ^a^	0.49 ± 0.17 ^b^	85.30 ± 8.82 ^ab^	0.19 ± 0.07 ^ab^
SE-40-M	4.27 ± 0.51 ^c^	0.25 ± 0.05 ^a^	0.69 ± 0.04 ^a^	75.22 ± 9.54 ^ab^	0.22 ± 0.03 ^ab^
SE-80-M	4.86 ± 0.57 ^c^	0.21 ± 0.02 ^a^	0.70 ± 0.11 ^a^	78.26 ± 8.80 ^ab^	0.25 ± 0.04 ^a^
SE-120-M	4.67 ± 1.03 ^c^	0.22 ± 0.04 ^a^	0.61 ± 0.01 ^ab^	63.15 ± 12.53 ^b^	0.25 ± 0.03 ^ab^
SE-160-M	5.54 ± 0.52 ^bc^	0.26 ± 0.04 ^a^	0.55 ± 0.04 ^ab^	81.02 ± 8.16 ^ab^	0.17 ± 0.02 ^b^
CK-B	15.06 ± 2.99 ^a^	0.36 ± 0.07 ^a^	0.42 ± 0.02 ^b^	238.23 ± 8.40 ^a^	0.12 ± 0.01 ^b^
pH-B	11.30 ± 1.96 ^b^	0.23 ± 0.07 ^b^	0.61 ± 0.16 ^a^	142.08 ± 6.20 ^b^	0.20 ± 0.07 ^a^
SE-40-B	7.86 ± 1.49 ^c^	0.24 ± 0.01 ^b^	0.39 ± 0.06 ^b^	72.64 ± 1.40 ^b^	0.13 ± 0.02 ^b^
SE-80-B	6.97 ± 1.23 ^c^	0.22 ± 0.06 ^b^	0.46 ± 0.09 ^ab^	75.35 ± 3.70 ^b^	0.15 ± 0.04 ^ab^
SE-120-B	6.03 ± 1.73 ^c^	0.28 ± 0.05 ^ab^	0.46 ± 0.05 ^ab^	82.80 ± 4.00 ^b^	0.13 ± 0.02 ^b^
SE-160-B	8.29 ± 0.68 ^bc^	0.24 ± 0.09 ^ab^	0.43 ± 0.06 ^b^	87.13 ± 2.30 ^b^	0.13 ± 0.01 ^b^

Note: Data are presented as mean ± SD (*n* = 6). Different superscript letters indicate significant differences (Tukey’s HSD test, *p* < 0.05). Comparisons were performed within each processing condition.

**Table 2:** Identification of Volatile Compounds Responsible for Fishy Odor in Rainbow Trout Using GC-MS Analysis.

Compounds	RI	RI *	Identification	Odor Description
Alcohols				
1-Penten-3-ol	670	688	RI, MS	grassy-green, vegetable, horseradish-like
1-Pentanol	764	764	RI, MS	fusel, bready, slightly fruity
1-Hexanol	864	865	RI, MS	green, herbaceous, woody, mildly fruity
1-Octen-3-ol	983	983	RI, MS	mushroom-like, earthy
Aldehydes				
Pentanal	686	690	RI, MS	green, bready, nutty
Hexanal	799	812	RI, MS	green, grassy, fatty
2-Hexenal (E)	855	850	RI, MS	green, leafy, fresh
Heptanal	902	913	RI, MS	fatty, citrus, green
2-Heptenal (E)	960	951	RI, MS	fatty, green, oily
Benzaldehyde	963	960	RI, MS	almond-like, cherry-like, sweet
Octanal	1003	1000	RI, MS	citrus, fruity, green
2-Octenal (E)	1048	1052	RI, MS	fatty, green, cucumber-like
2,6-Nonadienal (E,Z)	1161	1164	RI, MS	strong cucumber-like, green
Decanal	1262	1214	RI, MS	orange, citrus, waxy, sweet
2,4-Nonadienal (E,E)	1220	1210	RI, MS	fatty, cucumber/fried-like
2,4-Decadienal (E,E)	1353	1340	RI, MS	fried, fatty, deep-fried/chicken-like
E-14-Hexadecenal	–	–	RI, MS	oily, fresh-green, slightly sweet
Ketones				
Cyclohexanone	893	875	RI, MS	peppermint- or acetone-like
Hydrocarbons				
Isopropylcyclobutane	974	990.9	RI, MS	waxy
Cyclobutane (1-methylethylidene)-	997	998	RI, MS	waxy
3-Octyne	–	–	RI, MS	petroleum-like
Tetradecane	–	–	MS	mild, waxy, alkane-like
Pentadecane	–	–	MS	mild, waxy, oily

Note: RI is the retention index; experimentally determined RI values were rounded to the nearest integer, and RI values marked with an asterisk (*) were obtained from the NIST database.

**Table 3:** Regulation of Short-Chain and Long-Chain Aldehyde Distribution in Rainbow Trout by SE under marination and boiling conditions. SE-40, SE-80, SE-120, and SE-160 represent concentrations of 40, 80, 120, and 160 mg/mL, respectively. M and B indicate marination and boiling treatments, respectively.

	Hexanal (μg/kg)	Heptanal (μg/kg)	(E)-2-Heptenal (μg/kg)	1-Octen-3-ol (μg/kg)	Octanal (μg/kg)	Nonanal (μg/kg)
CK-M	963.27 ± 13.17 ^b^	148.02 ± 4.11 ^ab^	82.57 ± 2.98 ^a^	555.15 ± 3.32 ^a^	187.89 ± 4.17 ^a^	384.14 ± 8.59 ^ab^
pH-M	1025.42 ± 20.07 ^a^	136.80 ± 5.08 ^c^	64.18 ± 3.94 ^c^	492.24 ± 7.16 ^bc^	179.90 ± 2.44 ^a^	316.39 ± 4.18 ^d^
SE-40-M	649.92 ± 12.86 ^e^	133.98 ± 4.98 ^cd^	73.24 ± 4.52 ^b^	483.26 ± 6.07 ^c^	161.23 ± 8.79 ^b^	359.41 ± 3.13 ^c^
SE-80-M	837.04 ± 15.42 ^c^	140.23 ± 2.19 ^bc^	74.11 ± 3.14 ^b^	500.50 ± 8.18 ^bc^	149.49 ± 2.68 ^c^	379.64 ± 8.24 ^ab^
SE-120-M	763.88 ± 18.18 ^d^	152.96 ± 6.03 ^a^	63.39 ± 1.50 ^c^	515.90 ± 5.62 ^bc^	143.38 ± 4.15 ^c^	373.88 ± 6.36 ^b^
SE-160-M	960.28 ± 15.64 ^b^	127.26 ± 3.67 ^d^	62.55 ± 3.24 ^c^	530.55 ± 4.80 ^ab^	134.21 ± 3.66 ^d^	387.85 ± 5.78 ^a^
CK-B	637.36 ± 3.44 ^C^	239.94 ± 8.61 ^A^	-	-	75.42 ± 3.71 ^A^	135.05 ± 3.15 ^A^
pH-B	390.85 ± 12.75 ^E^	121.02 ± 9.73 ^C^	-	-	26.90 ± 3.89 ^DE^	78.39 ± 3.43 ^B^
SE-40-B	365.52 ± 22.20 ^E^	119.61 ± 9.15 ^C^	-	-	51.18 ± 4.69 ^B^	79.87 ± 2.16 ^B^
SE-80-B	688.61 ± 9.24 ^B^	142.49 ± 3.63 ^B^	-	-	22.51 ± 1.23 ^B^	74.36 ± 5.06 ^B^
SE-120-B	494.12 ± 19.20 ^D^	127.08 ± 7.08 ^C^	-	-	32.84 ± 3.00 ^CD^	133.41 ± 5.18 ^A^
SE-160-B	874.54 ± 20.77 ^A^	130.71 ± 8.66 ^BC^	-	-	37.21 ± 4.29 ^C^	134.87 ± 4.16 ^A^

Note: Data are presented as mean ± SD (*n* = 6). Different superscript letters indicate significant differences among groups. Uppercase and lowercase letters indicate statistical differences between different comparison groups (Tukey’s HSD test, *p* < 0.05).

## Data Availability

Data is contained within the article.

## Supplementary Materials

The following supporting information can be downloaded at https://www.mdpi.com/article/10.3390/gels12090845/s1, Table S1: Organic acid content, TPC, and pH of the SE; Table S2: Major Phenolic Compounds and Their Contents in *C. speciosa.*; Table S3: Changes in Protease Activity of SE under Marination Conditions. References [6,34,35,40,41,42,43,44] are cited in the supplementary materials.
