# Supplementary material for: Regulation of Rainbow Trout Muscle Myofibrillar Networks by Non-Enzymatic Bioactive Compounds from Chaenomeles speciosa
Source: Gels. 2026 Sep 16;12(9):845. doi: 10.3390/gels12090845 (PMC13606523; doi:10.3390/gels12090845)
Supplement: Supplementary file 1 [file gels-12-00845-s001.zip › gels-4505040-supplementary.pdf]

Supplementary Materials

Regulation of Rainbow Trout Muscle Myofibrillar Networks by Non-Enzymatic Bioactive Compounds from *Chaenomeles speciosa*

Rui Chai [1,2,3,4], Yidian Li [1,2,3,4], Zibo Song [5,6], Xuejiao Wang [1,2,3,4], Zhenna Zhang [7], Chaofan Guo [1,2,3,4,*] and Junjie Yi [1,2,3,4]

[1] Faculty of Food Science and Engineering, Kunming University of Science and Technology, Kunming 650500, China

[2] Innovation Research Group on Fruit and Vegetable Processing and Nutritional Health, Kunming 650500, China

[3] Yunnan Key Laboratory of Plateau Food Intelligent Processing, Kunming 650500, China

[4] Yunnan International Joint Laboratory of Green Food Processing, Kunming 650500, China

[5] Yunnan Provincial Key Laboratory of Applied Technology for Special Forest Fruits, Yuxi 653100, China

[6] Yunnan Maoduoli Group Food Co., Ltd., Yuxi 653100, China

[7] College of Food Science and Engineering, Northwest A&F University, Yangling 712100, China

* Correspondence: 20220031@kust.edu.cn

S1 Materials and methods

S1.1 Raw Materials and Chemicals

. All chemical reagents were of analytical grade and were purchased from Shanghai Aladdin Biochemical Technology Co., Ltd. (Shanghai, China).

S1.2 Preparation and Treatment of *C. speciosa* Aqueous Extract (SE)

C. speciosa was thoroughly washed and sliced according to previously described methods with slight modifications [1, 2]. Three separately weighed portions from the same batch of *C. speciosa* were used to prepare three independent extraction replicates. For each replicate, the slices were homogenized in deionized water at

a solid-to-liquid ratio of 1:10 *(w/v)*, followed by water-bath extraction at 60 °C for 30 min and ultrasonication at 30 kHz using an ultrasonic processor (XiaoMei Ultrasonic Instruments, Kunshan, China). Each extract was then centrifuged at 8,000 × g for 15 min using a refrigerated high-speed centrifuge (Multifuge X1 R, Thermo Fisher Scientific, Waltham, MA, USA), and the supernatant was collected separately. The three independently prepared aqueous extracts were freeze-dried separately under identical conditions within the same freeze-drying run using a vacuum freeze dryer (SCIENTZ-12N/C, Ningbo Scientz Biotech Co., Ltd., Ningbo, China) to minimize processing variability and ensure consistency in subsequent experiments. Each freeze-dried extract was subsequently re-dissolved in deionized water to obtain concentrations of 40, 80, 120, and 160 mg/mL for subsequent experiments.

S1.3.1 Determination of the Major Organic Acids and pH in *C. speciosa*

Extraction of Organic Acids: The extraction and analysis of organic acids were performed with minor modifications [3]. Freeze-dried sour papaya was homogenized with deionized water at a 1:10 (*w/v*) ratio. Then, 200 μL of $K_4[Fe(CN)_6]$ (15% *w/v*) and 200 μL of $ZnSO_4$ (30% *w/v*) were added, mixed thoroughly, and left to stand for 30 min. The mixture was centrifuged at 10,000 × g for 10 min at 4 °C, and the resulting supernatant was filtered through a 0.22 μm membrane before HPLC analysis.

Organic acids in the supernatant were determined using an HPLC system equipped with a Prevail Organic Acid column (250 mm × 4.6 mm, 5 μm). The injection volume was 30 μL, the column temperature was 25 °C, and the flow rate was 0.8 mL/min. Separation was performed under isocratic conditions with 25 mM potassium dihydrogen phosphate buffer (pH 2.5) as the mobile phase, and detection was carried out at 210 nm. All measurements were performed in triplicate.

pH Measurement: Sour papaya samples were homogenized with deionized water at a 1:2 (*w/v*) ratio. The pH of the resulting slurry was measured using a pH meter and recorded as the pH of the sour papaya slices. All measurements were performed in triplicate.

S1.3.2 Total phenolic content (TPC)Analysis of *C. speciosa*

The total phenolic content was determined using a slightly modified Folin–Ciocalteu colorimetric method [4]. The SE was mixed with 80% methanol at a ratio of 1:3 (*v/v*), vortexed thoroughly, and then centrifuged at 8000 × g for 10 min at 4°C. The supernatant was collected as the analytical solution, 100 μL of the supernatant was mixed with 500 μL of 10-fold diluted Folin–Ciocalteu reagent (diluted with distilled water) and allowed to stand for 5 min, followed by the addition of Na_2CO_3 (7.5% *w/v*) solution. The mixture was then incubated in the dark for 1 h, and the absorbance was measured at 765 nm. A standard curve was prepared using gallic acid, and the results were expressed as mg gallic acid equivalents per gram of fresh weight (mg GAE/g FW).

S1.3.3 LC–MS Analytical Method and Sample Preparation

Approximately 100 mg of sample was dissolved in methanol to 5 mL (20 mg/mL), followed by ultrasonic extraction for 10 min. The solution was centrifuged at 8000 × g for 15 min, and the supernatant was filtered through a 0.22 μm membrane prior to analysis.

LC–MS Conditions

Liquid chromatography–mass spectrometry analysis was performed using a Thermo Fisher LC–MS system (Thermo Fisher Scientific, Waltham, MA, USA) equipped with an electrospray ionization (ESI) source. Mass spectrometric detection was carried out in negative ion mode. The spray voltage was set at 3.5 kV, and the capillary temperature was maintained at 320 °C. Full scan MS data were acquired over an m/z range of 100–1000 with a resolution of 70,000, while MS/MS spectra were obtained at a resolution of 35,000.

Liquid Chromatography Conditions

Chromatographic separation was achieved using a Poroshell 120 EC-C18 column (2.1 mm × 100 mm, 1.9 μm). The column temperature was maintained at 35 °C, with an injection volume of 2 μL and a flow rate of 0.2 mL/min. The mobile phase consisted of 0.1% formic acid in water (A) and acetonitrile (B). The gradient elution program was as follows: 0–1 min, 5% B; 1–5 min, 15% B; 5–10 min, 38% B; 10–15 min, 65% B; 15–

18 min, 80% B; 18–20 min, 100% B; and 20–22 min, 100% B.

S1.4 Papain Activity Assay

Papain activity was determined using a neutral protease (NP) activity assay kit (Boxbio, Beijing, China) according to the manufacturer's instructions with minor modifications. For each sample, 1 mL of SE at different concentrations (40, 80, 120, and 160 mg/mL) was placed into a centrifuge tube. Each concentration was prepared with two treatments: boiled and marination.

Samples were processed by ice-bath sonication (300 W, 3 s on / 7 s off, total 3 min) and centrifuged at 8000 g for 10 min at 4 °C. The supernatant was collected for enzyme activity measurement. The enzymatic reaction was performed at 30 °C for 10 min, including 1 mL of sample supernatant, substrate solution, and standard solution as specified by the kit. Absorbance was measured at 680 nm using a spectrophotometer. The change in absorbance (Δ_A) was calculated by subtracting the blank reading from the sample reading.

Papain activity was calculated according to Equation (1):

$$NP\ activity\ \left(\frac{U}{mL}\right) = \frac{C_{standard} \times \Delta A_{sample} \times V_{total}}{\Delta A_{standard} \times V_{sample} \times T} \tag{1}$$

where $C_{standard}$ is the concentration of the standard acid solution (0.25 μmol/mL), V_{sample} is the volume of sample in the reaction, V_{total} is the total reaction volume, and T is the reaction time (10 min). One unit of enzyme activity was defined as the amount of enzyme catalyzing the formation of 1 μmol of acid per minute under the assay conditions. All measurements were performed in triplicate.

S2 Results and discussion

S2.1 Analysis of the Major Organic Acids and Total Polyphenol

In this work, the pH of the SE of sour papaya was determined to be 2.76, indicating a highly acidic profile consistent with that reported for other acidic fruit juices such as *Chaenomeles species*, whose juice pH typically ranges from approximately 2.5 to 2.8. The high acidity of the extract is consistent with the presence of abundant organic acids, which contribute substantially to the overall taste and chemical properties of the

sample [5, 6]. Organic acid profiling revealed that malic acid was the predominant organic acid in the water extract, followed by citric, tartaric, oxalic, and quinic acids. The dominance of malic acid aligns with previous studies on *C. speciosa* fruit, where malic acid and citric acid were identified as major organic acids in both aqueous and organic extracts, with malic acid accounting for a large proportion of the total organic acid content.

The high proportion of malic acid is consistent with its common role as a major organic acid in many fruits, contributing to sourness and being a key determinant of pH. The detection of citric acid and quinic acid also supports patterns observed in *Chaenomeles* and other fruit juices, where these acids, although present in lower concentrations than malic acid, contribute significantly to overall acid composition and taste characteristics [7].

In the work, the TPC of *C. speciosa* was determined to be 84.13 mg GAE/g FW. Previous studies have shown that *C. speciosa* is intrinsically a *C. species* with relatively high total phenolic content and strong antioxidant activity [4]. Therefore, our findings further support its potential value as a natural source of phenolic bioactive compounds.

Table S1 Organic acid content, TPC, and pH of the SE

Sample	pH	Citric acid (mg/g)	Quinic acid (mg/g)	Malic acid (mg/g)	Atranorin acid (mg/g)	TPC (mg GAE/g FW)
SE	2.76 ± 0.01	1.47±0.06	5.49±0.37	14.23±0.45	0.94±0.07	84.13±2.56

S2.2 LC–MS Analysis of Phenolic Compounds in *C. speciosa*

A total of nine compounds were identified, as shown in Table S2, all belonging to phenolic acids or flavonoids, suggesting the potential antioxidant capacity of the sample. Chlorogenic acid was the predominant compound (3.80 ± 0.45 mg/g FW), indicating its likely major contribution to the system. Catechin and neochlorogenic acid were present at relatively high levels, whereas 4-hydroxybenzoic acid and rutin were detected in much lower amounts (0.046 ± 0.003 and 0.012 ± 0.002 mg/g FW, respectively). Despite their low

concentrations, these compounds may still exert synergistic effects on the overall functionality.

Polyphenols are widely recognized for their antioxidant and anti-inflammatory activities. Moreover, they may interact with proteins via hydrophobic interactions, thereby modulating protein conformation and intermolecular interactions, which in turn could influence the stability and functional properties of the system.

Table S2 Major Phenolic Compounds and Their Contents in *C. speciosa*

Compound Name	Molecular Formula	Concentration (mg/g FW)	m/z	RT (min)	Reference Ion
4-Hydroxybenzoic acid	$C_7H_6O_3$	0.046 ± 0.003	137.02	2.25	[M-H]-1
Syringic acid	$C_9H_{10}O_5$	0.017±0.002	197.04	3.02	[M-H]-1
Chlorogenic acid	$C_{16}H_{18}O_9$	3.82 ± 0.45	353.08	5.42	[M-H]-1
Neochlorogenic acid	$C_{16}H_{18}O_9$	1.33 ± 0.22	353.08	5.71	[M-H]-1
Catechin	$C_{15}H_{14}O_6$	1.85 ± 0.13	289.07	6.63	[M-H]-1
4-Hydroxybenzaldehyde	$C_7H_6O_2$	0.140±0.009	121.02	6.55	[M-H]-1
Rutin	$C_{27}H_{30}O_{16}$	0.012 ± 0.002	609.14	8.64	[M-H]-1
Quercetin-3β-D-glucoside	$C_{21}H_{20}O_{12}$	0.022±0.002	463.08	9.24	[M-H]-1
4-Anisic acid	$C_8H_8O_3$	0.066±0.006	151.04	10.22	[M-H]-1

S3.1 Protease Activity of the SE

This work measured the protease activity in the SE. The results showed that only a low level of protease activity was detected in the marination aqueous extract. As the extract concentration increased from 40 mg/mL to 160 mg/mL, the enzyme activity increased from 2.10 U/mL to 2.85 U/mL. However, the overall enzyme activity remained low, and the increase was limited. When the extract concentration reached 120 mg/mL, the increasing trend of enzyme activity became gradual, and no significant difference was observed between the 120 mg/mL and 160 mg/mL groups. This indicates that the protease activity in the SE was not strong within this concentration range. Notably, no protease activity was detected in the aqueous extract after boiling, indicating that the protease components in the extract were sensitive to thermal treatment and could be inactivated by boiling. This is consistent with previous findings showing that papain, a plant thiol protease commonly used in food processing, undergoes thermal inactivation under elevated temperature conditions [8]. Since papain is a proteinaceous enzyme, high-temperature treatment may disrupt its spatial conformation and active site, thereby causing loss of catalytic activity.

However, based on the results of the rainbow trout experiment, the SE still showed certain effects on rainbow trout even after losing protease activity following boiling. This phenomenon suggests that the effects of the aqueous extract on rainbow trout may not be entirely dependent on papain or other protease-active components. Instead, its effects are more likely associated with heat-stable or non-enzymatic bioactive substances present in the aqueous extract.

Table S3 Changes in Protease Activity of SE under Marination Conditions

Concentration of SE (mg/mL)	40	80	120	160
Papain activity (U/mL)	2.10 ± 0.08^{c}	2.58 ± 0.06^{b}	2.77 ± 0.04^{a}	2.85 ± 0.03^{a}

Note: Different superscript letters in each column imply that mean values are significantly different (Tukey's HSD test, $p < 0.05$).

References

[1] Hou, M.; Lin, C.; Ma, Y.; Shi, J.; Liu, J.; Zhu, L.; Bian, Z. One-Step Enrichment of Phenolics from *Chaenomeles Speciosa* (Sweet) Nakai Fruit Using Macroporous Resin: Adsorption/Desorption Characteristics, Process Optimization and UPLC-QqQ-MS/MS-Based Quantification. *Food Chemistry*, **2024**, *439*, 138085. https://doi.org/10.1016/j.foodchem.2023.138085.

[2] Lopeda-Correa, M.; Valdés-Duque, B. E.; Osorio-Tobón, J. F. Ultrasound-Assisted Extraction of Phenolic Compounds from Adenaria Floribunda Stem: Economic Assessment. *Foods*, **2022**, *11* (18). https://doi.org/10.3390/foods11182904.

[3] Zhang, S.; Yin, Y.; Ya, X.; Xiang, Y.; Song, Z.; Deng, Y.; Guo, C.; Devahastin, S.; Yi, J. Physicochemical Properties, Instrumental Taste and Aroma Changes of Thermally and Non-Thermally Pasteurized *Chaenomeles Sinensis* Slices upon Processing and Storage. *LWT*, **2026**, *242*, 119106. https://doi.org/10.1016/j.lwt.2026.119106.

[4] Liu, Z.; Esveld, E.; Vincken, J.-P.; Bruins, M. E. Pulsed Electric Field as an Alternative Pre-Treatment for Drying to Enhance Polyphenol Extraction from Fresh Tea Leaves. *Food Bioprocess Technol*, **2019**, *12* (1), 183–192. https://doi.org/10.1007/s11947-018-2199-x.

[5] Hellín, P.; Vila, R.; Jordán, M. J.; Laencina, J.; Rumpunen, K.; Ros, J. M. Characteristics and Composition of Chaenomeles Fruit Juice; 2003.

[6] Marat, N.; Danowska-Oziewicz, M.; Narwojsz, A. Chaenomeles Species—Characteristics of Plant, Fruit and Processed Products: A Review. *Plants (Basel)*, **2022**, *11* (22), 3036. https://doi.org/10.3390/plants11223036.

[7] Zheng, X.; Wang, H.; Zhang, P.; Gao, L.; Yan, N.; Li, P.; Liu, X.; Du, Y.; Shen, G. Chemical Composition, Antioxidant Activity and α-Glucosidase Inhibitory Activity of Chaenomeles Speciosa from Four Production Areas in China. *Molecules*, **2018**, *23* (10). https://doi.org/10.3390/molecules23102518.

[8] Katsaros, G. I.; Katapodis, P.; Taoukis, P. S. High Hydrostatic Pressure Inactivation Kinetics of the Plant Proteases Ficin and Papain. *Journal of Food Engineering*, **2009**, *91* (1), 42–48. https://doi.org/10.1016/j.jfoodeng.2008.08.002.
